# Evaluating the Performance of Seven Ongoing Satellite Altimetry Missions for Measuring Inland Water Levels of the Great Lakes

**DOI:** 10.3390/s22249718

**Published:** 2022-12-12

**Authors:** Zhiyuan An, Peng Chen, Fucai Tang, Xueying Yang, Rong Wang, Zhihao Wang

**Affiliations:** 1 College of Geomatics, Xi’an University of Science and Technology, Xi’an 710054, China; 2 State Key Laboratory of Geodesy and Earth’s Dynamics, Innovation Academy for Precision Measurement Science and Technology, CAS, Wuhan 430077, China; 3 Beijing Key Laboratory of Urban Spatial Information Engineering, Beijing 100045, China

**Keywords:** satellite altimetry, inland water level, performance

## Abstract

Satellite altimetry can provide long-term water level time series for water bodies lacking hydrological stations. Few studies have evaluated the performance of HY-2C and Sentinel-6 satellites in inland water bodies, as they have operated for less than 1 and 2 years, respectively. This study evaluated the measured water level accuracy of CryoSat-2, HY-2B, HY-2C, ICESat-2, Jason-3, Sentinel-3A, and Sentinel-6 in the Great Lakes by in-situ data of 12 hydrological stations from 1 January 2021 to 1 April 2022. Jason-3 and Sentinel-6 have the lowest mean root-mean-square-error (RMSE) of measured water level, which is 0.07 m. The measured water level of Sentinel-6 satellite shows a high correlation at all passing stations, and the average value of all correlation coefficients (R) is also the highest among all satellites, reaching 0.94. The mean RMSE of ICESat-2 satellite is slightly lower than Jason-3 and Sentinel-6, which is 0.09 m. The stability of the average deviation (bias) of the ICESat-2 is the best, with the maximum bias only 0.07 m larger than the minimum bias. ICESat-2 satellite has an exceptionally high spatial resolution. It is the only satellite among the seven satellites that has retrieved water levels around twelve stations. HY-2C satellite has the highest temporal resolution, with a temporal resolution of 7.5 days at station 9075014 in Huron Lake and an average of 10 days in the Great Lakes region. The results show that the seven altimetry satellites currently in operation have their own advantages and disadvantages, Jason-3 and Sentinel-6 have the highest accuracy, ICESat-2 has higher accuracy and the highest spatial resolution, and HY-2C has the highest temporal resolution, although it is less accurate. In summary, with full consideration of accuracy and space-time resolution, the ICESat-2 satellite can be used as the benchmark to achieve the unification of multi-source data and establish water level time series.

## 1. Introduction

About 3% of the world’s land surface is covered by lakes [1]. These lakes provide freshwater resources necessary for the survival of aquatic and terrestrial organisms, and are also the main source of fresh water for various human activities, drinking, and irrigation [2,3]. The temporal and spatial changes in lake water levels record the changes in the regional ecological environment to a certain extent, and are an important indicator to reflect the local climate [4]. In recent decades, the spatial distribution of Earth’s water resources and the process of surface water circulation have undergone immense changes, triggering a series of ecological crises [5]. Therefore, long-term dynamic monitoring of lake water levels is particularly important. As a traditional method for monitoring lake water levels, hydrological stations are only set up in some large lakes due to political and economic reasons [6,7]. It cannot effectively monitor lakes with harsh environments and remote locations, such as most lakes located on the Qinghai-Tibet Plateau. Therefore, there is an urgent need for a long-term and effective method for monitoring the water levels of lakes around the world without relying on hydrological stations. To obtain a long-term and effective method for monitoring global lake water levels, many studies have attempted to use physical models to estimate lake levels around the world [8,9]. However, due to the significant differences in physical factors in different regions, the existing physical models can only estimate the water level for a particular region [10]. The maturity of satellite altimetry technology only compensates for this defect. As an essential method to obtain inland water levels rather than hydrological stations, it can eliminate national and regional restrictions and conduct long-term series observations on the global and specific regions.

Satellite altimetry has been widely used to monitor water levels in inland lakes since the first altimetry satellite (Geodetic/Geophysical) was placed into operation in 1985. There have been more than thirteen satellite radar altimetry missions in the past three decades [11]. Existing altimetry satellites can be divided into radar and laser altimetry satellites. Most altimetry satellites with radar altimeters operate in traditional low-resolution mode (LRM). The SAR mode and SARin mode have a smaller footprint than the traditional low-resolution mode (LRM) and can obtain water levels of smaller water bodies. CryoSat-2 satellite, launched in 2010, can switch between three operating modes: LRM, SAR, and SARin [12]. Sentinel-3A satellite, launched in 2016, was the first to operate in SAR mode globally [13]. HY series satellite is China’s first marine dynamic environmental satellite constellation integrating active and passive microwave remote sensing. Sentinel-6, the follow-up mission of Jason-1, Jason-2, and Jason-3, is the first satellite carrying Poseidon-4 into orbit. Poseidon-4 uses a 9 kHz pulse repetition frequency (PRF), which is ~4 times greater than Jason-3. Poseidon-4 uses an interleaved radar chronogram to ensure that the synthetic aperture radar (SAR) mode and low-resolution mode (LRM) are performed at the same time. The interleaved (open burst) transmit and receive approach indicates that twice the number of samples are available compared with the Copernicus Sentinel-3 radar altimeter bringing a notable improvement in altimeter noise characteristics [14]. ICESat-2 satellite is equipped with a laser altimeter. Compared with the radar altimeter, the laser altimeter has the characteristics of high precision, high sampling density, and small footprint [15].

The accuracy of inland water level retrieval from different altimetry satellites in different areas and environments has been evaluated by many studies. Birkinshaw, O’Donnell [16] compared the water level of the Mekong River obtained by ERS-2 and Envisat satellites with the measured data, and the obtained RMSEs were between 0.44–0.65 m and 0.46–0.76 m, respectively, which proved the feasibility of satellite altimetry in establishing a unified river hydrological model. Villadsen, Deng [17] proposed the multiple waveform persistent peak (MWaPP) retracker based on CryoSat-2 satellite, and the RMSE in two medium-sized lakes (Lake Vanern in Sweden and Lake Okeechobee in Florida) and Amazon River were between 9.1 and 29.0 cm. Jiang, Schneider [11] discussed all the important steps in the workflow for hydrologic analysis with CryoSat-2. Xue, Liao [18] proposed the novel and improved MWaPP retracker (ImpMWaPP) and verified it based on the in-situ data of seven lakes on the Qinghai-Tibet Plateau, and the measured water level accuracy in the ice-free period of Qinghai Lake reached 0.08 m. Liu, Yao [19] evaluated a concentrated probability density function (PDF) method using in-situ data from 12 lakes in China. The results show that PDF can accurately observe the above lake water levels, and the obtained average RMSE and average R are 0.27 m and 0.84, respectively. Li, Li [20] used ICESat-1, Envisat, and Sentinel-3A from 2002–2017 to monitor lake levels in the middle and lower reaches of the Yangtze River in China over a long period. Xiang, Li [15] evaluated the accuracy of ICESat-2 and GEDI satellites over the Great Lakes and the Mississippi River. ICESat-2 satellite still acquired high accuracy (RMSE averaged 0.06 m in the Great Lakes and 0.12 m in the Mississippi River).

The above studies demonstrate the accuracy of previously launched altimetry satellites in inland waters. HY-2C and Sentinel-6 are critical for maintaining the continuity of inland water measurements and also represent important data sources for improving the spatial resolution of measurements. The current validity of Sentinel-6 and HY-2C data is less than 1 and 2 years, respectively. The literature on the evaluation of Sentinel-6 and HY-2C is very limited, and few studies have compared these two satellites with previously launched satellites. Therefore, the overall goal of this study is to verify the accuracy of the measured water level from the seven satellites. Moreover, we aim to evaluate and compare the performance of seven altimetry satellites in the Great Lakes, analyze the factors that affect the accuracy of altimetry satellites to measured water levels, and explore the potential to monitor dynamic changes in inland water levels. This lays the foundation for establishing long-term high spatial resolution inland water levels using multi-source altimetry data.

## 2. Materials and Methods

### 2.1. Study Area

**Figure 1:**
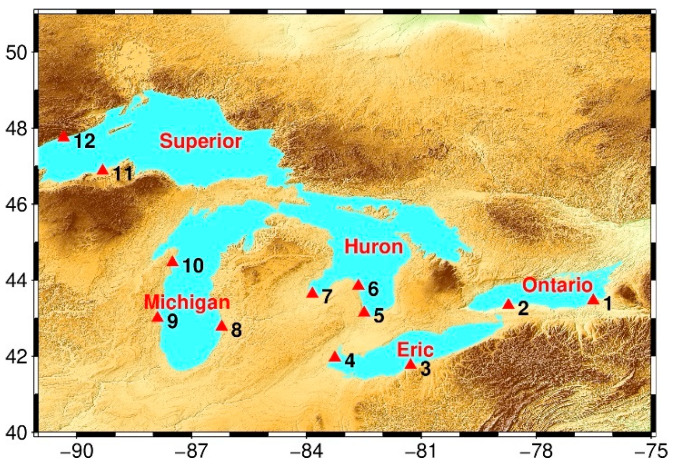
Location of water bodies and surface hydrological stations in the study area. Light blue areas are water bodies, and red triangles are locations of hydrological stations.

The study area is the Great Lakes of North America, which is the largest group of freshwater lakes in the world. The total area of the Great Lakes is 245,200 km^2^, of which the United States accounts for 72%, and Canada accounts for 28%. The full water storage is about 22.8 trillion cubic meters, accounting for about 20% of the total freshwater lakes in the world. The Great Lakes are subject to few climatic extremes and can provide a stable hydrological environment, making it an excellent place to assess the accuracy of satellite measurements of water levels [21]. Moreover, many hydrological stations in this area provide a large amount of in-situ data for evaluating the measured water level by satellite. Furthermore, CryoSat-2, HY-2B, HY-2C, ICESat-2, Jason-3, Sentinel-3A, and Sentinel-6 satellites all pass through this area; therefore, they are excellent water bodies to evaluate the accuracy of measured water levels in temporal and spatial resolutions. The specific details of the research area in this study are shown in Figure 1. The water body boundary is obtained from the Global Lakes and Wetlands Database (GLWD) database (https://www.worldwildlife.org/, accessed on 1 September 2021). A red triangle represents the location of hydrological station. The detailed information of hydrological station is shown in Table 1.

### 2.2. Data

#### 2.2.1. Altimetry Satellite Data

**Figure 2:**
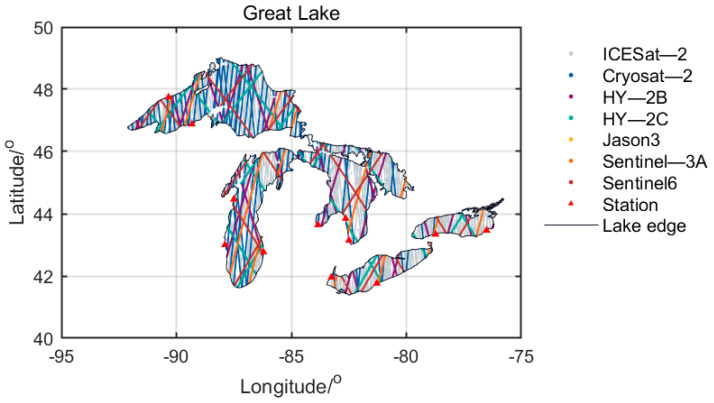
Satellite track distribution map.

This study selects six radar altimetry satellites (CryoSat-2, HY-2B, HY-2C, Jason-3, Sentinel-3A, and Sentinel-6) and a laser altimetry satellite (ICESat-2), and uses their data in the Great Lakes region from January 2021 to March 2022. In this study, the calculated results of the above data are compared, and the in-situ data are used to verify the measured water level accuracy. The satellite trajectory distribution can be seen in Figure 2. The data details of the seven satellites are shown in Table 2.

ESA launched CryoSat-2 satellite on 8 April 2010. CryoSat-2 was equipped with the most advanced synthetic aperture interferometric radar altimeter (SIRAL) in the world at that time, and three operating modes (low-resolution mode (LRM), synthetic aperture radar (SAR), and synthetic aperture radar interferometer (SARin)) were designed internally [22]. CryoSat-2 is the first altimetry mission to provide an orbital footprint of 300 m, and its reference ellipsoid is WGS84 [17]. This study uses the LRM_L2 data downloaded from CryoSat-2 Science Server. HY-2B was launched in October 2018, HY-2C satellite was launched in September 2020, and TOPEX ellipsoid was used as the elevation reference. HY-2B satellite is mainly equipped with dual-frequency altimeters (Ku and C bands), with a revisit period of 14 days at the initial stage of operation and a revisit period of 168 days at the end of the mission [23]. The coverage rate of HY-2C scatter meter in the global sea area is not less than 90%. The revisit period in the early stage of the operation is 10 days, and the orbit revisit period will become 400 days when the mission is about to end. The sensor geophysical data records (SDR) data used in this article can be downloaded from the China Ocean Satellite Data Service System.

As a follow-up mission to ICESat-2 satellite, ICESat-2 carries the advanced terrain laser altimeter (ATLAS) with WGS84 as the elevation reference. ATLAS has a total of six laser beams, divided into three pairs, and each beam pair laser consists of a strong energy beam and a weak energy beam (the number of photons is about 4:1) [24]. Dandabathula and Srinivasa Rao [25] evaluated the performance of the strong and weak beams of ICESat-2 satellite over 15 reservoirs in India and found that the strong beam was 7% more accurate than the weak beam measurements. This study uses the strong energy beam data of ALT13 of ICESat-2, which can be downloaded from the NSIDC website.

Jason-3 was launched on 17 January 2016, carrying Poseidon-3B radar altimeter, using the TOPEX ellipsoid as the elevation reference [26]. This study selects the highest precision final product geophysical data records (GDR) data from Jason-3, which can be downloaded from the National Centers for Environmental Information (NCEI). Sentinel-3A, the world’s first altimetry satellite operating in SAR mode globally, uses WGS84 as the elevation reference. This study uses the “enhanced” file of the NTC product of Sentinel-3A’s SAR_2_LAN. The data can be downloaded from the Copernicus Open Access Hub. Sentinel-6 satellite, a follow-up mission of Jason-3, carried the Poseidon-4 radar altimeter and microwave radiometer, with WGS84 as the elevation reference. This study used NTC data from Sentinel-6’s level 2 low-resolution (LR_2), which can be downloaded from the European Meteorological Satellite Applications Organization (EUMETSAT).

#### 2.2.2. In-Situ Data

The National Oceanic and Atmospheric Administration (NOAA) provides monthly water level data and tide data for US lakes, as well as hourly water level reports and daily water level reports for the Great Lakes. In-situ data from twelve stations in the Great Lakes were used in this study to evaluate the performance of seven satellites in the measured water levels. These stations use the International Great Lakes Datum of 1985 (IGLD85) as the vertical reference.

#### 2.2.3. Lake Water Level Extraction

This study first uses the Global Lakes and Wetlands Database (GLWD) mask data to extract the vector boundaries of the Great Lakes, and then obtains the data located inside the lakes according to the vector boundaries [27]. Due to the large area of the Great Lakes, there may be significant differences in water levels even in different regions of the same lake (a comparison of the in-situ data of different stations found that there is an average deviation of 2–5 cm between different stations in the same lake). Therefore, in this study, the satellite data located within 0.4° around the station and inside the lake were acquired with the station as the center, this radius can guarantee both sufficient altimetry observations and small fluctuations of the lake surface. The data were considered as the valid data inside the lake. After using this method to divide the effective area, each station can be regarded as a small independent lake; therefore, the results obtained in this study are also applicable to other inland water bodies. The satellite measurement of the lake level can be determined as given in Equation (1):

(1) $$H=H_{\mathrm{alt}}-R_{\mathrm{range}}-\left(\mathrm{iono}+\mathrm{wet}+\mathrm{dry}+\mathrm{solid}+\mathrm{pole}\right)-\mathrm{Geoid}$$

where H is the lake level, $H_{\mathrm{alt}}$ is the distance from the satellite to the reference ellipsoid, $R_{\mathrm{range}}$ is the distance between altimeter and Earth’s surface, iono is the ionospheric correction (this study uses the GIM global ionospheric model), wet is the wet tropospheric correction, dry is the dry tropospheric correction, solid is the solid earth tide correction, pole is the polar tide correction, and geoid is used to change the vertical datum from the reference ellipsoid to the geoid (this study uses the EGM2008 geoid model). The details of the above correction are shown in Table 3.

**Figure 3:**
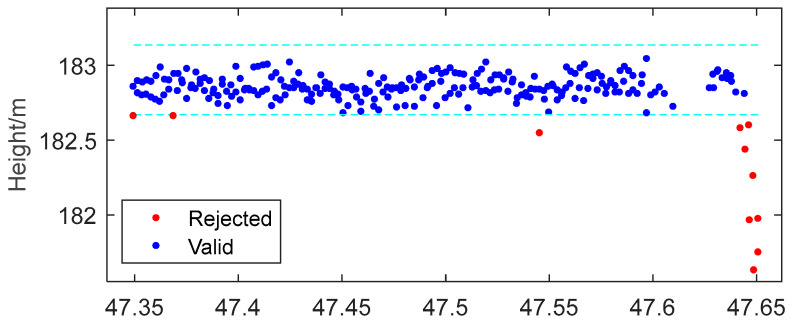
Sentinel-6 satellite outlier detection example over the 9099090 station. The result shows valid (blue) and rejected (red) water levels. The confidence limit is the cyan dotted line.

CryoSat-2 directly obtains the water level through the parameter (height_1_20_ku). The ICESat-2 satellite does not require any additional processing. The water levels of HY-2B, HY-2C, Jason-3, Sentinel-3A, and Sentinel-6 satellites are all calculated by Equation (1), and then valid data are obtained according to the quality control annotations in Table 4. Due to the hooking effect and the influence of land contamination, there will be a large error [30]. This study took the median of the water level as the representative and three times the standard deviation as the confidence limit, and then calculated the average effective water level as the satellite measured water level of the day. As can be seen in Figure 3, all heights influenced by land contamination and hooking effect are detected as outliers and the remaining heights represent a flat surface.

#### 2.2.4. Unified Satellite Measured Water Level Datum

Due to the different reference ellipsoids used by different satellites, the vertical datum used by the satellite to retrieve the water level is also different from the in-situ data. Therefore, it is necessary to convert the measured water level of the satellite and the in-situ data to the same datum after extracting the water level from the altimetry satellite. Jason-3, HY-2B, and HY-2C use T/P as a reference ellipsoid. This study converts T/P to WGS84, and the T/P reference ellipsoid is 0.707 m larger than the WGS84 ellipsoid in North America [31]. The vertical conversions from WGS84 to NAVD88 and from IGLD85 to NAVD88 were performed using the datum transformation tool (VDatum). CryoSat-2, ICESat-2, Sentinel-3A, and Sentinel-6 satellites convert WGS84 to NAVD88 datums.

#### 2.2.5. Outlier Detection of Water Level in Satellite Retrieval

Affected by various near-shore environments, the satellite measured water level will inevitably have abnormal values; therefore, it is necessary to eliminate the noise of the measured water level. This study uses the support vector regression (SVR) to identify and eliminate abnormal water levels. SVR is an advancement of the support vector machine (SVM) [32], which is used as a classification algorithm for applications, such as pattern recognition and machine learning. Depending on the mathematical problem, the kernel for the regression varies. One can use linear, polynomial, or radial base functions [33]. Since the water level contains both seasonal and trend changes, this study uses the radial base function to perform the regression [34], and then takes the two times rate of change (*diff*) in the water level time series as the confidence limit to separate valid and invalid water levels.

(2) $$\mathrm{diff}=\frac{\sum_{i=2}^{N}h_{i}-h_{i-1}}{N-1}$$

(3) lim=2∗diff

where *N* is the number of water levels, *h_i_* is the *i*-th water level, and *lim* is the confidence limit.

**Figure 4:**
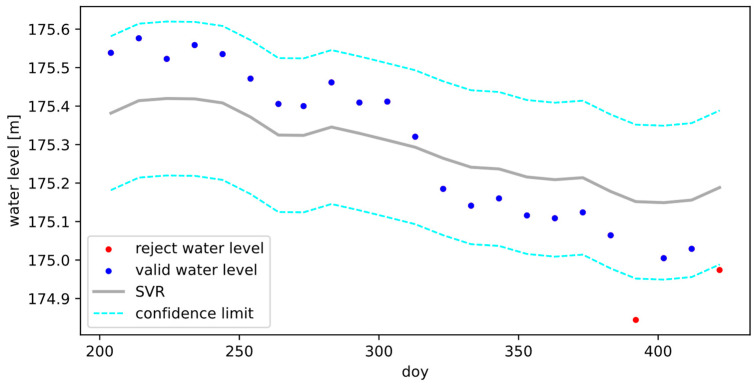
Example of an applied SVR on the time series of 9087068 station. The result shows valid (blue) and rejected (red) water levels. The confidence limit is the cyan dotted line.

Figure 4 shows the results of an applied SVR on the time series of 9087068 station. The gray solid line is the regression function estimated using the SVR radial vector basis function. The cyan dotted line is the confidence limit. The blue and red points are the valid and rejected water levels, respectively. Each rejected water level height represents one complete satellite overflight.

#### 2.2.6. Validation Indicators

In this study, the performance of CryoSat-2, HY-2B, HY-2C, ICESat-2, Jason-3, Sentinel-3A, and Sentinel-6 satellites for inland measured water levels is compared. The root-mean-square-error (RMSE), correlation coefficient (R), and mean deviation (Bias) are used as indicators to evaluate the accuracy of measured water level (HY-2B and HY-2C do not use the retracking algorithm, whereas CryoSat-2, Jason-3, Sentinel-3A, and Sentinel-6 all use the ocean retracking algorithm). In addition to accuracy, the spatial-temporal resolution of the satellite altimetry mission was critical for inland water level monitoring [35]. Higher temporal resolution can capture short-term changes and more detailed information for global inland water level monitoring. In this study, the number of measured water levels (N) is used as an indicator of the temporal resolution of altimetry satellites. Since the 12 stations of the Great Lakes are located at different locations, the more stations around the satellite have measured water levels, the higher the spatial resolution of the satellite. Therefore, the number of satellites passing through the station is used as the index of spatial resolution, as shown in the following equations:

(4) $$RMSE=\sqrt{\frac{1}{m}\sum_{i=0}^{m}{\left(H_{measured}-H_{gauge}\right)}^{2}}$$

(5) $$Bias=\frac{1}{m}\left(H_{measured}-H_{gauge}\right)$$

where *m* is the total number of satellites that overpass a lake during the study period, *H_measured_* is the lake water level estimate by a satellite on date *i*, *H_gauge_* is the in-situ gauge measurement on date *i*.

## 3. Results and Discussion

The RMSE represents the accuracy of the lake measured water level compared with the in-situ data. A smaller RMSE indicates that the measured water level has a small random deviation and a higher precision. Table 5 shows the RMSE of satellite measured water levels over the Great Lakes. CryoSat-2 has a lower RMSE (0.06 m) than the other satellites at station 9099044. Villadsen, Deng [17] proposed MWaPP retracker based on CryoSat-2 satellite, and the RMSE in two medium-sized lakes (Lake Vanern in Sweden and Lake Okeechobee in Florida) and Amazon River were between 9.1 and 29.0 cm, which were comparable to this study. The RMSEs of HY-2B and HY-2C were between 0.04–0.23 m and 0.07–0.26 m, respectively. The RMSE of ICESat-2 ranged from 0.05 to 0.14 m, with the highest accuracy at stations 9063090, 9075014, and 9087068. Xiang, Li [15] used ICESat-2 satellite to obtain water levels in the Great Lakes region with RMSEs ranging from 0.04–0.14 m, which is similar to this study. Jason-3 satellite RMSE is between 0.04 and 0.14 m, and has not achieved the highest accuracy among the seven satellites at any station; however, the average RMSE is lower and comparable to Sentinel-3A and Sentinel-6. Sentinel-3A has the lowest RMSE around four stations (9052030, 9075002, 9075014, 9087031), and the lowest RMSE obtained around station 9087031, which is 0.04 m. Nielsen, Andersen [13] validated the performance of Sentinel-3A satellite in more than 100 lakes in the United States and Canada. Since no benchmark transformation was performed, the median RMSE obtained using ocean retracker was 0.25 m, which is significantly lower than this study. Xiang, Li [15] evaluated Sentinel-3A performance in 15 North American lakes during 2016–2017. After subtracting the average deviation between the observed water level and the measured water level, the obtained RMSE is between 3.55 and 7.62 cm; with the exception that the RMSE obtained by Sentinel-6 satellite at station 9087068 is 0.14 m, and the other stations are not higher than 0.10 m. Among the seven altimetry satellites, Jason-3 and Sentinel-6 have the lowest mean RMSE at 0.07 m. HY-2C had the highest mean RMSE, which is 0.15 m.

R represents the ability of the satellite to calculate water level changes, and a higher R indicates that the satellite can well capture water level changes. In this study, R > 0.8 is highly correlated, 0.6 < R < 0.8 is middle correlated, R < 0.6 is low correlated. Table 6 presents the R of satellite measured water levels over the Great Lakes. The CryoSat-2 satellite has low correlations at stations 9087031 and 9087068, and shows high correlations around the other stations. HY-2B satellite shows a low correlation around 9087068 station, and the rest of the stations are highly correlated. The measured water level of HY-2C has a high correlation as a whole, and only the correlation of station 9063053 in Erie Lake is between 0.6 and 0.8, showing a medium correlation. The remaining five stations showed a high correlation between the measured water level and the in-situ data. The overall R of ICESat-2 satellite is relatively high, and the average R reaches 0.86. Jason-3 satellite showed an extremely high correlation except around station 9052076 where the correlation was low, which is 0.69, and in five stations the correlation reached above 0.95. The correlation between the measured water level of Sentinel-3A satellite and the in-situ data shows a two-level differentiation trend. Among the nine passing stations, the R of four stations reached more than 0.95, and the R of two stations was less than 0.6. The correlation around station 9063053 in Erie Lake is the lowest of all stations for all satellites, which is 0.18. Sentinel-6 satellite showed a high correlation at all passing stations. Moreover, the average R was the highest among all satellites, which is 0.94. In general, the correlation coefficient of water level obtained by ICESat-2 satellite and radar altimetry satellite is equivalent, and there is no low correlation.

**Figure 5:**
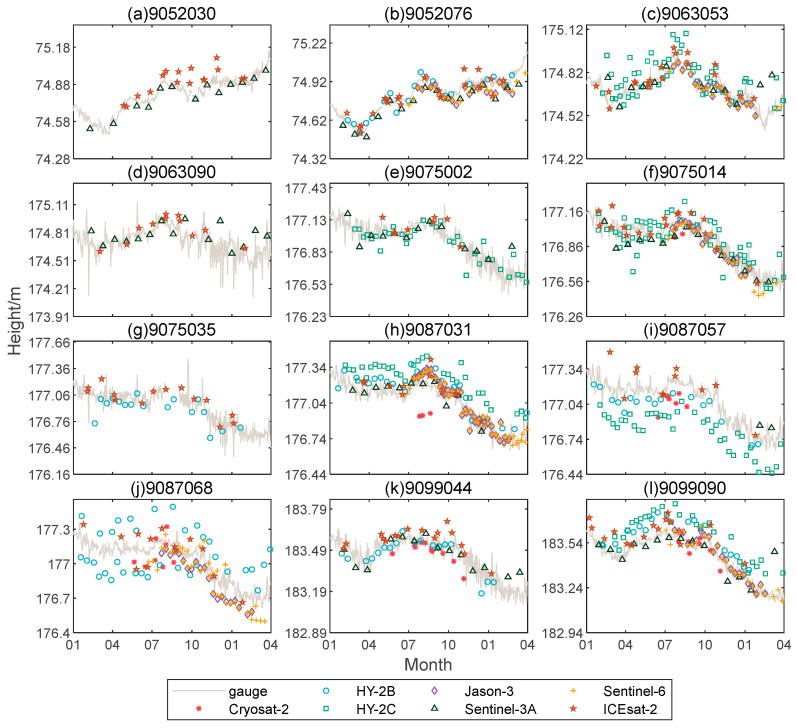
Time series comparison of satellite measured water level and the in-situ data around 12 stations in the Great Lakes from 1 January 2021 to 1 April 2022.

Bias indicates the average deviation between the satellite measured water level and the in-situ data. A lower bias represents that the satellite-derived water level has higher accuracy, while a relatively stable bias represents that the measured water level is less disturbed by external factors. It can be seen from Table 7 that the overall deviation of CryoSat-2 is the largest, and the maximum bias is 0.25 ± 0.03 m. HY-2B has a higher bias at station 9099090, and the absolute values of the other bias are all less than 0.11 m. The bias of HY-2C at 9075002 and 9075014 stations is 0. Both Jason-3 and Sentinel-6 satellites obtained relatively stable bias values, higher than Sentinel-3A by 0.03 m. Among the seven satellites, the bias of ICESat-2 satellite is the most stable, and the maximum bias is only 0.07 m larger than the minimum bias. Sentinel-3A satellite has the smallest mean value of bias at 0.03 m. However, compared with ICESat-2, the variability is larger. Therefore, it is recommended to use ICESat-2 satellite as the reference datum, when the multi-source altimetry data are unified to the same satellite datum according to the method of Chen and Liao [34]. The reasons are as follows:

(1) The deviation between the measured water level by ICESat-2 satellite and the in-situ data is stable.
(2) ICESat-2 satellite has a high spatial resolution and can obtain the measured water level in most lakes and provide offset corrections for other satellites.
(3) It can be seen from Figure 5 that ICESat-2 satellite does not have the same situation as HY-2B satellite at station 9087068: The measured water level is distributed above and below the in-situ data, RMSE is higher and R is lower than the other satellites, but the bias value is the smallest among all satellites, which is only 0.03 m.

The higher the temporal resolution of the satellite, the more times it passes around the station. It can be seen from Table 8 that CryoSat-2 satellite has the lowest temporal resolution among the seven satellites, with only three observations at the 9075014 and 9087031 stations. HY-2B has a higher temporal resolution, with 35 observations at the 9087068 station. The time resolution of HY-2C satellite at five stations is higher than the other satellites, and represents the highest temporal resolution of all stations at station 9075014 in Huron Lake. The overall temporal resolution of ICESat-2 satellite is low, with only nine observations in 9075002 station. Jason-3 provides a high temporal resolution, with an average of 25 observations per station. The temporal resolution of Sentinel-3A is comparable to ICESat-2. Moreover, Sentinel-6 satellite provides a high temporal resolution around the six stations it passes through, second only to HY-2C, with an average of 35 observations per station. All satellites have passed through stations 9087031 and 9099090, and the average temporal resolution over the entire study period is 2.3 and 3.2 days, respectively. Only two satellites passed through station 9063090, and the average temporal resolution over the entire study period is 15.1 days. The above results show that it is possible to obtain the global inland water level with the high temporal resolution based on the fusion of multi-source altimetry data.

Spatial resolution refers to the minimum distance between two adjacent objects identified on remote sensing images. Due to the fact that the distance between the stations selected in this study is significantly large, the spatial resolution of satellite cannot be directly expressed. Therefore, the number of satellites passing through the station is used to verify the spatial resolution indirectly. It can be seen from Table 8 that the number of hydrological stations for CryoSat-2, HY-2B, HY-2C, ICESat-2, Jason-3, Sentinel-3A, and Sentinel-6 is 6, 7, 6, 12, 6, 9, and 6, respectively. ICESat-2 satellite has the highest spatial resolution, followed by Sentinel-3A, and the other satellites are equal. The lidar altimetry satellite ICESat-2 provides water levels at 12 stations with the highest spatial resolution.

To more clearly reflect the relationship between measured water level and the in-situ data, a comparison figure of satellite measured water level and the in-situ data around 12 stations of the Great Lakes was drawn. It can be seen from Figure 5 that around stations 9063053, 9075014, 9087068, and 9099090, there are clear deviations between satellite measured water level and the in-situ data from January to April. The reason for this phenomenon may be that the low temperature in winter causes a large area of ice on the surface of the lake; wrong retracking points will be identified during the retracking process, resulting in large deviations between the satellite observed water level and the actual measured water level. Shu, Liu [35] detailed the effect of winter lake ice on satellite measured water levels, and proposed a bimodal correction algorithm, which can accurately estimate the water level of lakes covered by lake ice in winter by retrieving lake ice thickness. Although the algorithm is not used for correction in this study to compare the measurement accuracy of seven satellites, it plays an important role in establishing long-term high-precision water level time series.

The advantages of multi-satellite measured water level can be clearly seen at the 12 stations. For example, if only one altimetry satellite is used to acquire the water level time series, there will be no observation of water level around at least three stations, except for ICESat-2 satellite. If multiple sources of altimetry data are used to obtain the water level time series, there will be no stations without observed water levels.

**Figure 6:**
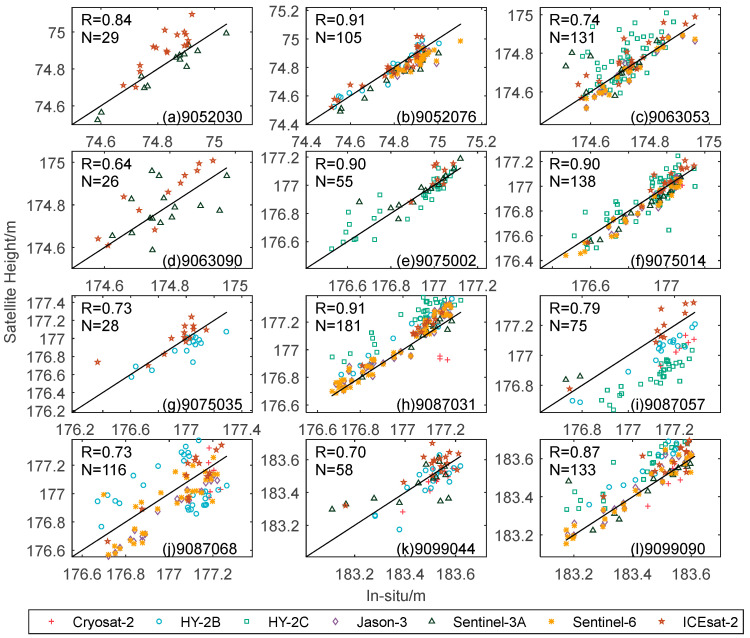
Comparison of the correlation between satellite measured water levels and in-situ data around 12 stations in the Great Lakes, where R is the total correlation coefficient, and N is the total number of measured water levels.

To illustrate the correlation between measured water levels and the in-situ data, the correlation figure of the satellite measured water level and the in-situ data around 12 stations of the Great Lakes was drawn. As can be seen from Figure 6 and Table 6, the measured water levels from the correlation coefficient of the seven satellites are high with the in-situ data. Among them, the correlation coefficient of 9052076 station is the highest, which is 0.92. The correlation coefficient of 9063090 station is the lowest, which is 0.63. Among the 12 stations, the measured water level around the seven stations has a high correlation with the in-situ data, and the rest are moderately correlated, and there is no low or weak correlation. This shows that the fusion of multi-source altimetry data has great potential in capturing detailed changes in water level. Only ICESat-2 and Sentinel-3A satellites pass through station 9052030, and their correlation coefficients are 0.94 and 0.98, but their overall correlation coefficient is only 0.90, which is lower than the respective correlation coefficients of ICESat-2 and Sentinel-3A satellites. In general, the measured water level obtained by the seven satellites has a high correlation with the in-situ data, and only a few stations are low; for example, Sentinel-3A at station 9063053.

In summary, ICESat-2 satellite has the following advantages and disadvantages compared with the other six radar satellites: (1) The accuracy of ICESat-2 satellite is comparable to Jason-3 and Sentinel-3A satellites, and higher than the other radar altimetry satellites, which can meet the needs of daily measurement. (2) ICESat-2 satellite has a higher spatial resolution than the other radar altimeter satellites due to its dense orbit. (3) The time resolution in the same area is significantly lower than the radar altimeter satellite. CryoSat-2 satellite has a low spatial and temporal resolution due to its different orbital operation mode.

**Figure 7:**
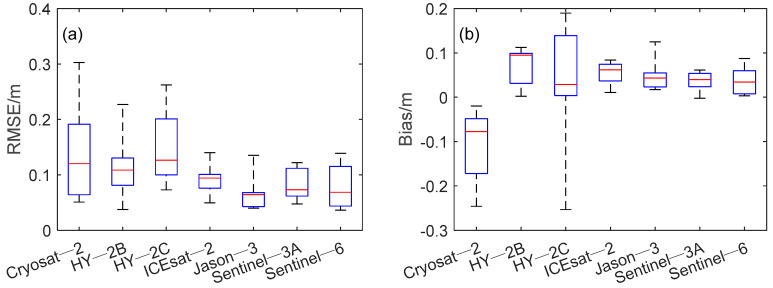
Box plot of RMSE (**a**) and bias (**b**) between seven altimetry measured water levels and in-situ data for the Great Lakes during all acquisition times. The top and bottom of each box show the 25th and 75th percentiles of the RMSE and bias, and the red line in the middle of the box shows the median (50th percentile). The two whiskers extend to the farthest outlying RMSE and bias represents the maximum and minimum values of RMSE and bias.

Figure 7 shows the RMSE and bias comparisons of seven altimetry satellites over the Great Lakes. It can be clearly seen that the RMSE and bias obtained by ICESat-2, Jason-3, Sentinel-3A, and Sentinel-6 satellites are generally lower than those obtained by CryoSat-2, HY-2B, and HY-2C satellites. HY-2C satellite has the largest RMSE and the lowest accuracy. The fluctuation degree of the ICESat-2 satellite deviation is low, and measured water levels that agree relatively well with the in-situ data can be obtained. The RMSE of Sentinel-6 is comparable to Jason-3 and slightly higher than Sentinel-3A. The bias value obtained by the Sentinel-6 satellite is lower, but the variability is higher than ICESat-2 satellite. The RMSE of ICESat-2 is second only to Jason-3 and Sentinel-6, and bias is the most stable of all satellites.

## 4. Conclusions

This study discusses the accuracy of Sentinel-6, HY-2C, and other satellites in measuring inland water level, and evaluates their performance. First, seven altimetry satellite missions CryoSat-2, HY-2B, HY-2C, ICESat-2, Jason-3, Sentinel-3A, and Sentinel-6 are used to calculate the water level around twelve hydrologic stations in the Great Lakes of North America from 1 January 2021 to 1 April 2022. The water level time series of the unified datum is obtained using the vertical datum conversion and gross error elimination. Then, an evaluation is carried out through different accuracy indicators, such as RMSE, R, bias, temporal resolution, and spatial resolution. The following conclusions were drawn in the Great Lakes region:

(1) ICESat-2 satellite is second only to Jason-3 and Sentinel-6 satellites in accuracy among the seven satellites, with the highest spatial resolution and the best overall performance. Therefore, it can be used as a datum satellite for multi-source satellite altimetry measured water levels.
(2) As a follow-up mission to Jason-3, Sentinel-6 satellite has the highest accuracy among the seven satellites. Sentinel-6 is superior to Sentinel-3A in capturing water level changes and temporal resolution, and Sentinel-3A is superior to Sentinel-6 in spatial resolution.
(3) HY-2C has the highest temporal resolution among all satellites, and the number of measured water levels by a single satellite around stations 9063053 and 9075014 is equivalent to the total number of measured water levels by the other six satellites. Although HY-2B and HY-2C are slightly lower than the other satellites, the overall accuracy of HY-2C has a certain improvement compared with HY-2B satellite. It can be expected that the follow-up satellites of the HY series will achieve higher accuracy. Moreover, HY-2C satellite provides the highest temporal resolution at the same station, and has great potential for capturing more detailed water level data.

Based on the above studies, it can be found that satellite altimetry has great potential in inland measured water levels. The upcoming launch of Sentinel-6B and SWOT satellites will provide important data sources for inland water level retrieval. Among them, SWOT satellite will be equipped with a new Ka-band radar interferometer (KaRIn) for the first time, which is expected to achieve higher accuracy in inland water level measurement.

## Figures and Tables

**Table 1:** Table of details of hydrological stations in the study area used as accuracy verification.

Location	No.	Station ID	Lon (°)	Lat (°)
Ontario Lake	1	9052030	−76.51	43.47
	2	9052076	−78.73	43.34
Erie Lake	3	9063053	−81.28	41.76
	4	9063090	−83.26	41.96
Huron Lake	5	9075002	−82.49	43.14
	6	9075014	−82.64	43.85
	7	9075035	−83.85	43.64
Michigan Lake	8	9087031	−86.21	42.77
	9	9087057	−87.89	43.00
	10	9087068	−87.50	44.46
Superior Lake	11	9099044	−89.32	46.88
	12	9099090	−90.34	47.75

**Table 2:** Details of the selected satellites and their data in the study.

Mission	Duration in This Study	Data Level	Data Type	Repeat Cycle (Day)	Along-Track Interval (m)
CryoSat-2	2021.1–2022.2	Level 2	LRM	369 (subcycle 30)	300
HY-2B	2021.1–2022.3	Level 2	SDR	14	less than 2000
HY-2C	2021.1–2022.3	Level 2	SDR	10	less than 2000
ICESat-2	2021.1–2022.3	ATL13	ALT13	91	17
Jason-3	2021.7–2022.2	Level 2	GDR	10	2000–4000
Sentinel-3A	2021.1–2022.3	Level 2	SAR	27	300
Sentinel-6	2021.6–2022.3	Level 2	LR	10	300

**Table 3:** Sources of the main parameters for altimetry measurement.

	Jason-3, Sentinel-3A, and CryoSat-2	HY-2B and HY-2C	ICESat-2	Sentinel-6
retracker	ocean	/	/	ocean
iono	GIM	GIM	/	GIM
wet	European Center for Medium Range Weather Forecasting (ECMWF)	National Centers for Environmental Prediction (NCEP)	/	ECMWF
dry	ECMWF	NCEP	/	ECMWF
solid	Cartwright and Edden [28]	Cartwright and Edden [28]	/	Calculated using Cartwright and Tayler tables
pole	Wahr [29]	Wahr [29]	/	Calculated using Desai model
geoid	EGM2008	EGM2008	EGM2008	EGM2008

**Table 4:** Parameters and quality control marks used in satellite measured water level.

Mission	Water Level	Quality Control
CryoSat-2	height_1_20_ku	/
HY-2B	alt_20hz range_20hz_ku	surface_type = 1
HY-2C	alt_20hz range_20hz_ku	surface_type = 1
ICESat-2	ht_water_surf	/
Jason-3	alt_20hz range_20hz_ku	surface_type = 1 qual_alt_1hz_range_ku = 0
Sentinel-3A	alt_20_ku range_ocean_20_ku	surface_type_20_ku = 1
Sentinel-6	altitude range_ocean	/

**Table 5:** RMSE of seven altimetry satellites at twelve stations in the Great Lakes (unit: m).

Lake	Station ID	CryoSat-2	HY-2B	HY-2C	ICESat-2	Jason-3	Sentinel-3A	Sentinel-6
Ontario	9052030	/	/	/	0.10	/	0.05	/
	9052076	/	0.04	/	0.05	0.07	0.07	0.07
Erie	9063053	/	/	0.10	0.07	0.04	0.12	0.04
	9063090	/	/	/	0.09	/	0.11	/
Huron	9075002	/	/	0.07	0.08	/	0.07	/
	9075014	0.09	/	0.10	0.06	0.06	0.06	0.07
	9075035	/	0.13	/	0.14	/	/	/
Michigan	9087031	0.30	0.11	0.20	0.10	0.07	0.04	0.06
	9087057	0.19	0.11	0.26	0.12	/	0.13	/
	9087068	0.16	0.23	/	0.10	0.14	/	0.14
Superior	9099044	0.06	0.07	/	0.09	/	0.12	/
	9099090	0.05	0.12	0.15	0.10	0.04	0.09	0.04
Mean		0.14	0.12	0.15	0.09	0.07	0.09	0.07

**Table 6:** Correlation coefficient R of seven altimetry satellites at twelve stations in the Great Lakes.

Lake	Station ID	CryoSat-2	HY-2B	HY-2C	ICESat-2	Jason-3	Sentinel-3A	Sentinel-6
Ontario	9052030	/	/	/	0.86	/	0.97	/
	9052076	/	0.97	/	0.94	0.69	0.97	0.87
Erie	9063053	/	/	0.71	0.89	0.95	0.18	0.97
	9063090	/	/	/	0.90	/	0.40	/
Huron	9075002	/	/	0.92	0.74	/	0.85	/
	9075014	0.94	/	0.83	0.95	0.96	0.99	0.98
	9075035	/	0.87	/	0.81		/	/
Michigan	9087031	−0.70	0.97	0.93	0.92	0.97	0.96	0.98
	9087057	0.93	0.97	0.95	0.87		1	/
	9087068	0.29	0.34	/	0.88	0.99	/	0.88
Superior	9099044	0.89	0.83	/	0.77		0.66	/
	9099090	0.89	0.96	0.94	0.80	0.97	0.75	0.97
Mean		0.54	0.85	0.88	0.86	0.92	0.77	0.94

**Table 7:** Bias of seven altimetry satellites at twelve stations in the Great Lakes (unit: m).

Lake	Station ID	CryoSat-2	HY-2B	HY-2C	ICESat-2	Jason-3	Sentinel-3A	Sentinel-6
Ontario	9052030	/	/	/	0.08 ± 0.06	/	−0.03 ± 0.03	/
	9052076	/	0 ± 0.04	/	0.01 ± 0.05	−0.05 ± 0.04	−0.06 ± 0.03	−0.06 ± 0.03
Erie	9063053	/	/	0.05 ± 0.09	0.04 ± 0.05	−0.02 ± 0.03	0.05 ± 0.11	−0.03 ± 0.03
	9063090	/	/	/	0.07 ± 0.06	/	0 ± 0.11	/
Huron	9075002	/	/	0.00 ± 0.07	0.05 ± 0.07	/	0.02 ± 0.07	/
	9075014	−0.07 ± 0.01	/	0.01 ± 0.10	0.03 ± 0.05	−0.04 ± 0.05	−0.05 ± 0.03	−0.05 ± 0.04
	9075035	/	−0.10 ± 0.09	/	0.07 ± 0.12	/	/	/
Michigan	9087031	−0.25 ± 0.03	0.10 ± 0.04	0.19 ± 0.06	0.08 ± 0.05	0.05 ± 0.05	00.1 ± 0.04	0.04 ± 0.05
	9087057	−0.17 ± 0.03	−0.10 ± 0.04	−0.25 ± 0.05	0.06 ± 0.09	/	/	/
	9087068	−0.09 ± 0.12	0.05 ± 0.22	/	0.03 ± 0.10	−0.12 ± 0.04	0.09 ± 0.03	−0.08 ± 0.11
Superior	9099044	−0.05 ± 0.04	−0.03 ± 0.07	/	0.06 ± 0.07	/	0.01 ± 0.14	/
	9099090	−0.02 ± 0.05	0.11 ± 0.03	0.14 ± 0.05	0.08 ± 0.05	0.02 ± 0.04	0 ± 0.09	0.01 ± 0.04
Mean		−0.11 ± 0.06	0.07 ± 0.10	0.10 ± 0.07	0.06 ± 0.07	0.05 ± 0.04	0.03 ± 0.07	0.04 ± 0.06

**Table 8:** The number N of measured water levels by seven altimetry satellites at twelve stations in the Great Lakes.

Lake	Station ID	CryoSat-2	HY-2B	HY-2C	ICESat-2	Jason-3	Sentinel-3A	Sentinel-6	total
Ontario	9052030	/	/	/	18	/	13	/	33
	9052076	/	19	/	22	22	16	28	110
Erie	9063053	/	/	58	20	20	12	23	138
	9063090	/	/	/	14	/	15	/	30
Huron	9075002	/	/	34	9	/	13	/	44
	9075014	3	/	60	18	19	14	28	149
	9075035	/	15	/	13	/	/	/	33
Michigan	9087031	3	21	33	12	45	11	57	197
	9087057	6	17	39	11	/	2	/	81
	9087068	8	35	/	13	21	/	47	139
Superior	9099044	10	18	/	16	/	14	/	62
	9099090	9	15	31	15	22	14	28	144
Mean		6.5	20	42.5	15	25	12	35	

## Data Availability

The authors gratefully acknowledge the data distribution agencies who provided the publicly released data used in this work. The CryoSat-2 data were obtained from CryoSat-2 Science Server (https://science-pds.cryosat.esa.int/, accessed on 1 July 2022). The HY-2B/C data were obtained from National Satellite Ocean Application Service (http://www.nsoas.org.cn/, accessed on 13 July 2022). The GLAS and ATLAS data were provided by the National Snow and Ice Data Center website (https://nsidc.org/data, accessed on 15 July 2022). The Jason-3 data were obtained from National Centers for Environmental Information (https://www.ncei.noaa.gov/, accessed on 5 July 2022). The Sentinel-3A data were obtained from Copernicus Open Access Hub (https://scihub.copernicus.eu/, accessed on 1 September 2022). The Sentinel-6 data were obtained from the European Organization for the Application of Meteorological Satellites (https://www.eumetsat.int/, accessed on 1 August 2022). The in-situ data of the Great Lakes were provided by the National Oceanic and Atmospheric Administration (https://tidesandcurrents.noaa.gov/map/index.html, accessed on 1 June 2022). The water mask of the Great Lakes was provided by Global Lakes and Wetlands Database (https://www.worldwildlife.org/, accessed on 1 September 2021).
